# Designing Human-Centered AI to Prevent Medication Dispensing Errors: Focus Group Study With Pharmacists

**DOI:** 10.2196/51921

**Published:** 2023-12-25

**Authors:** Yifan Zheng, Brigid Rowell, Qiyuan Chen, Jin Yong Kim, Raed Al Kontar, X Jessie Yang, Corey A Lester

**Affiliations:** 1 Department of Clinical Pharmacy College of Pharmacy University of Michigan Ann Arbor, MI United States; 2 Department of Industrial and Operations Engineering College of Engineering University of Michigan Ann Arbor, MI United States

**Keywords:** artificial intelligence, communication, design methods, design, development, engineering, focus groups, human-computer interaction, medication errors, morbidity, mortality, patient safety, safety, SEIPS, Systems Engineering Initiative for Patient Safety, tool, user-centered design methods, user-centered, visualization

## Abstract

**Background:**

Medication errors, including dispensing errors, represent a substantial worldwide health risk with significant implications in terms of morbidity, mortality, and financial costs. Although pharmacists use methods like barcode scanning and double-checking for dispensing verification, these measures exhibit limitations. The application of artificial intelligence (AI) in pharmacy verification emerges as a potential solution, offering precision, rapid data analysis, and the ability to recognize medications through computer vision. For AI to be embraced, it must be designed with the end user in mind, fostering trust, clear communication, and seamless collaboration between AI and pharmacists.

**Objective:**

This study aimed to gather pharmacists’ feedback in a focus group setting to help inform the initial design of the user interface and iterative designs of the AI prototype.

**Methods:**

A multidisciplinary research team engaged pharmacists in a 3-stage process to develop a human-centered AI system for medication dispensing verification. To design the AI model, we used a Bayesian neural network that predicts the dispensed pills’ National Drug Code (NDC). Discussion scripts regarding how to design the system and feedback in focus groups were collected through audio recordings and professionally transcribed, followed by a content analysis guided by the Systems Engineering Initiative for Patient Safety and Human-Machine Teaming theoretical frameworks.

**Results:**

A total of 8 pharmacists participated in 3 rounds of focus groups to identify current challenges in medication dispensing verification, brainstorm solutions, and provide feedback on our AI prototype. Participants considered several teaming scenarios, generally favoring a hybrid teaming model where the AI assists in the verification process and a pharmacist intervenes based on medication risk level and the AI’s confidence level. Pharmacists highlighted the need for improving the interpretability of AI systems, such as adding stepwise checkmarks, probability scores, and details about drugs the AI model frequently confuses with the target drug. Pharmacists emphasized the need for simplicity and accessibility. They favored displaying only essential information to prevent overwhelming users with excessive data. Specific design features, such as juxtaposing pill images with their packaging for quick comparisons, were requested. Pharmacists preferred accept, reject, or unsure options. The final prototype interface included (1) checkmarks to compare pill characteristics between the AI-predicted NDC and the prescription’s expected NDC, (2) a histogram showing predicted probabilities for the AI-identified NDC, (3) an image of an AI-provided “confused” pill, and (4) an NDC match status (ie, match, unmatched, or unsure).

**Conclusions:**

In partnership with pharmacists, we developed a human-centered AI prototype designed to enhance AI interpretability and foster trust. This initiative emphasized human-machine collaboration and positioned AI as an augmentative tool rather than a replacement. This study highlights the process of designing a human-centered AI for dispensing verification, emphasizing its interpretability, confidence visualization, and collaborative human-machine teaming styles.

## Introduction

Medication errors present a significant health care safety challenge with notable implications worldwide. In the United States, the Food and Drug Administration receives roughly 100,000 medication error–related reports annually [1]. It is estimated that medication errors cost up to US $42 billion each year and account for 5%-6% of all hospitalizations worldwide [2,3]. Despite the role of pharmacists in medication verification for detecting and mitigating medication errors, dispensing errors still occur in around 1.5% of all prescriptions [4].

Medication verification is the process of visually confirming the medication bottle contents match the prescribed medication before dispensing the medication to a patient. Dispensing errors are a mismatch between the prescribed and dispensed medications. Pharmacy staff have used tools, such as barcode scanning and double-checks, to decrease and detect dispensing errors [5,6]. Nevertheless, these methods have their limitations: the barcode on medication packages may not accurately represent the medications dispensed, and barcode scanning can lead to workarounds and policy deviations [7,8]. Human double-checks can introduce new errors, including miscommunication or misinterpretation of information, and are influenced by cognitive limitations such as fatigue, distractions, and inattentiveness [9,10]. Therefore, human cognition may not be best used for a vigilance task, including medication dispensing verification. These limitations highlight the need for innovative solutions.

Artificial intelligence (AI) has the potential to assist with the pharmacy verification process, as it can recognize pills using computer vision, thus overcoming the limitations of indirect barcode scanning checks [11]. AI can also analyze data more rapidly and identify errors that human pharmacists might miss [12]. However, the successful implementation of AI depends on its capacity to effectively collaborate with health care professionals to improve clinical outcomes and support accurate clinical decision-making [13]. The challenge is to build human-centered AI, communicate AI outputs to users effectively, and build appropriate trust, ensuring consistent decision-making over long periods of time [14].

The Systems Engineering Initiative for Patient Safety (SEIPS) 3.0 and the Human-Machine Team (HMT) theoretical frameworks may guide researchers to design systems that integrate AI with human expertise. The SEIPS model, grounded in the principles of human-centered systems engineering, commonly known as human factors and ergonomics, paints a comprehensive picture of how work systems influence health-related outcomes. Specifically, it sheds light on how different factors in the system can directly or indirectly affect patient safety. This model serves as an invaluable tool for researchers to dive deep into the nuances of system engineering to better understand its implications for patient safety and make necessary adjustments for improvement [15-17]. It encompasses 6 primary elements: person, tasks, tools and technologies, physical environment, organizational conditions, and external environment. These components interact in complex ways to influence both processes and outcomes in health care settings. Successfully applied to medication safety, it can help elucidate interactions between health care professionals and technology to enhance patient safety [7,18-21]. The HMT framework is a cornerstone when considering the integration of AI in health care settings. While the current wave of research and media attention surrounding AI in health care seems preoccupied with evaluating AI systems’ capability to substitute human cognitive and clinical judgment, the HMT framework pivots the perspective toward viewing AI as a teammate rather than a replacement. This shift is vital because a replacement-centric view can inadvertently lead to apprehensions among health care professionals. They might start perceiving AI systems as competitors, potentially undermining their authority and expertise. Such perceptions could culminate in resistance to AI adoption, hindering its potential benefits in patient care [22,23]. To fully use the capabilities of AI in health care, it is essential to promote a cooperative teaming style where AI systems and health care professionals work together, each amplifying the strengths of the other. The HMT framework can be the groundwork for this synergistic collaboration.

The objective of this study is to develop an interpretable, human-centered AI system that can seamlessly integrate within pharmacies, earn pharmacists’ trust, and aid their decisions so that dispensing errors are minimized. While various technologies have historically been designed to aid the verification process, little is known about how AI can specifically bolster the pharmacist dispensing verification process. In this paper, we aim to identify the challenges pharmacists encounter during medication dispensing and explore how a human-centered AI system can team up with pharmacists to prevent medication dispensing errors based on qualitative focus groups.

## Methods

### Recruitment and Participants

Purposive sampling was used during the recruitment and screening of the participants. Pharmacists were recruited through email listservs for professional pharmacists at the University of Minnesota College of Pharmacy and the University of Wisconsin School of Pharmacy. An email was sent to the listserv managers asking the managers to post an institutional review board (IRB)–approved flyer about the study. Interested pharmacists were instructed to email the study team to schedule a screening phone call. During the screening call, a research team member provided detailed information about the study, and interested individuals were asked the following three screening questions: (1) Are you at least 18 years old? (2) Do you have pharmacy practice experience? If so, what is/was your experience? (3) Do you have access to a computer with high-speed internet and a web camera? Eligible participants were invited to attend a focus group session. Before the initial focus group session, eligible participants reviewed, signed, and dated a prospective agreement information sheet that described the details of the study, their role as participants, and who to contact with questions about the study and enrollment. A total of 10 pharmacists completed the screening, 9 signed the prospective agreement form, and 8 agreed to participate in the focus groups. Individuals who were unable to attend the first round were ineligible to participate in subsequent rounds. Focus groups were conducted on the internet in 3 rounds from September 2021 to July 2022.

### Focus Group Design

#### Overview

**Figure 1:**
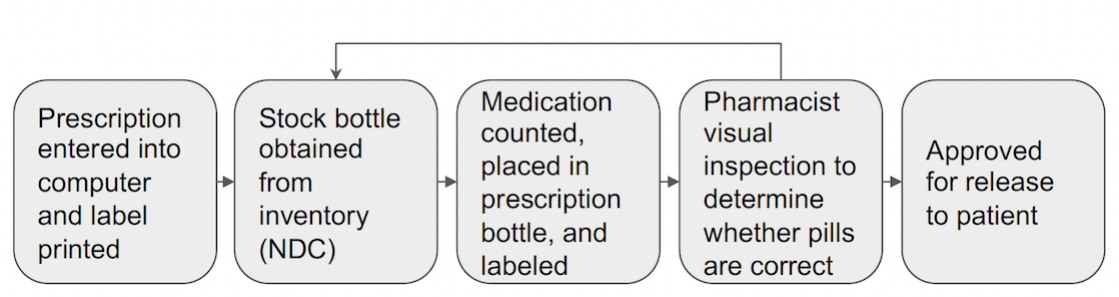
The current medication verification process. Pharmacy staff enter the prescription information into a computer, generating a label. The drug is picked up from inventory using the NDC, counted, and placed into a labeled prescription bottle. A pharmacist conducts a visual inspection to ensure the correct pills are dispensed before approving the medication for release to the patient. NDC: National Drug Code.

A multidisciplinary research team including a licensed pharmacist, pharmacy researchers, health care human factor experts, and engineers led the focus groups. Before focus group 1, the research team developed Figure 1 to help elicit feedback on the challenges and problems faced during the dispensing process related to product verification. As Figure 1 shows, the verification process involves 5 steps, from prescription entry to drug release approval. Any detected errors trigger prescription denial and preparation reiteration. Internet-based verification parallels the in-person process but uses camera images of prescription vials instead of a physical examination.

To ensure productive discussions and maximize participation, each round of focus groups was divided into 2 sessions. These sessions were conducted on the internet, lasting 2 hours each. Each round of the focus groups targeted specific objectives. Interview guides for each focus group are listed in Multimedia Appendices 1-3.

#### Focus Group 1

In focus group 1, participants were introduced to the envisioned human-AI collaboration, problem identification, and solution brainstorming. During this session, pharmacists explored the current practice of medication verification, including their roles, tasks, responsibilities, challenges encountered, and existing supportive features. The facilitator introduced the concept of internet-based verification, prompting pharmacists to share their experiences. In the second section, participants brainstormed solutions for human-AI collaboration aimed at enhancing the safety and effectiveness of medication image verification. Each participant was sent a link to a Google Slide (Google Inc) containing a sample verification screen containing a reference image, fill image, and prescription information. The facilitator provided a brief overview of how to use Google Slide to mark up and modify the template. Written instructions were also included in the slide deck. Participants were asked to independently create mockups of an interface incorporating their desired features for the AI help element as well as their preferred layout. Participants were asked to present their mockup to the group and discuss the rationale for each element and the layout.

#### Focus Group 2

In focus group 2, participants provided feedback on the prototype model and discussed potential implementation scenarios in pharmacy practice. In this session, pharmacists were introduced to the HMT framework, emphasizing concepts such as transparency (observability and predictability), augmenting cognition (directing attention, exploring the solution space, and adaptability), and coordination (directability, calibrated trust, and common ground). They were encouraged to consider these concepts as the research team presented a prototype AI system. Subsequently, pharmacists provided feedback on our prototype and discussed its features in relation to the HMT framework. The group also examined the following three implementation scenarios: (1) AI supervising the pharmacist, (2) pharmacist supervising the AI, and (3) pharmacist auditing the AI. Trust in the system, factors influencing trust, and design modifications to enhance trustworthiness were discussed.

Before focus group 3, our team developed a prototype AI interface to assist pharmacists with dispensing verification tasks. Addressing the challenge of verifying pills during dispensing, we used a Bayesian neural network that predicts the dispensed pills’ National Drug Code (NDC). The Bayesian neural network is realized by applying the random dropout technique to the ResNet-34 neural network [24-26]. Instead of providing 1 prediction for the probabilities of belonging to a specific NDC, the Bayesian network provides a set of possible probabilities, which can be used in turn to quantify uncertainty. In this study, the model sampled 50 possible probabilities for each image. The NDC with the highest mean probability was selected as the predicted NDC based on the dispensing pill image.

#### Focus Group 3

In focus group 3, the third prototype iteration was presented, and pharmacist feedback was gathered for further refinement. This session involved five main tasks: (1) model description, (2) communicating model output to pharmacists, (3) interactive user interface design, (4) discussion of the experimental design for the project’s second phase, and (5) a pill substitution activity. Pharmacists completed a pick-and-place design activity to explore multiple methods of presenting AI model output to pharmacists. Participants were exposed to possible design elements and then grouped into teams of 2 or 3. Each team was given access to a Google Slide file containing the design elements and placed in Zoom (Zoom Video Communications, Inc) breakout rooms. They spent 8-10 minutes creating their preferred user interface, focusing on the most efficient and user-friendly arrangement for dispensing verification. Subsequently, the teams regrouped to showcase their interfaces and discuss their selections. The session concluded with a review of the model’s errors and a discussion about the experimental design for the subsequent simulated environment testing of the AI system.

### Data Collection and Analysis

Professional transcriptions were created from the audio recordings after each session and reviewed by the research team. An independent team member conducted a content analysis based on the SEIPS and HMT theoretical frameworks, and other team members reviewed the coding with any discrepancies addressed collectively [27-29]. After revisions, the primary results were summarized and presented in tables, figures, and a separate final prototype visualization.

### Ethical Considerations

The IRB of the University of Michigan determined this research is exempt from IRB oversight under exemption 3 of 45 CFR 46.104(d)(3) (IRB00001995) [30]. Participants electronically signed and dated a prospective research agreement before the first focus group. The prospective agreement informed participants that the focus group sessions would be video recorded through Zoom and the recordings would be transcribed without identifiers. The Zoom recordings are accessible only by IRB-approved study team members and will be deleted at the end of the study. Participants received US $100 for each focus group session they attended, with a total compensation of up to US $300.

## Results

### Characteristics of the Participants

We initially enrolled 9 pharmacists; however, due to scheduling conflicts, 1 participant withdrew, resulting in 8 participants across the first 2 focus groups and 7 in the third. Among the study participants, 7 provided comprehensive demographic information. Their average age was 39 (SD 11) years, comprising 3 female and 4 male participants. When considering racial identity, 4 participants identified as White, 1 as Asian, 1 as Native Hawaiian or Pacific Islander, and 1 as multiracial.

### Medication Dispensing Verification Process and Challenges

A total of 8 pharmacists participated in the initial focus groups that discussed medication verification processes and identified challenges. One pharmacist had experience with internet-based verification.

Through the SEIPS interaction models [17], we dissected key task characteristics influencing the typical pharmacy setting verification process (Table 1). Technological aids include optical and barcode systems, yet limitations arise from dispensing software constraints, such as only accepting a single NDC for multi-NDC prescriptions. Task-related aspects encompass bottle opening, label double-checking, NDC number verification, and pill comparison with reference images. Personal and environmental challenges include pharmacists’ visual capabilities, pill size similarity, external distractions, interruptions, and heavy workloads. Organizational hurdles include high staff turnover and extended shifts, potentially escalating error risks. Pharmacists underlined the role of experience, technological familiarity, intuition, and trust in technicians for efficient verification. However, continuous disruptions and long shifts can strain cognitive capacities, adversely impacting error detection. Medication verification challenges also stem from pill resemblance in size and shape, limited human visual distinction, and the absence of standardized pill appearances in the United States. This leads to difficulty differentiating similar packaging and imprints across various dosage strengths. To improve identification, pharmacists often use magnification and enhanced lighting.

**Table 1:** Medication verification task characteristics based on the Systems Engineering Initiative for Patient Safety (SEIPS) model for the following categories: tasks (TA), person (P), environment (E), technology and tools (TE), organization (O), external environment (EE).

Medication verification work system components			Characteristics
**Tasks^a^**			
	TA1	Memorizing the color, shape, size, and marking of pills from the reference	
	TA2	Opening prescription vials to see pills	
	TA3	Checking the marking, color, shape, and quantity of pills and matching them with the reference pill	
	TA4	Checking for the correct label	
**Person**			
	P1	Visual ability to distinguish pills	
	P2	Vigilance and focus maintenance (fatigue and long shifts)	
	P3	Mental checklists	
	P4	Comfort level with new technologies	
	P5	Trust in coworkers	
	P6	Availability of coworkers	
**Environment^b^**			
	E1	Common distractions and disruptions	
	E2	High volume of prescriptions to verify	
	E3	Multiple NDCs^c^ for a single drug product	
	E4	Multitasking	
**Technology and tools^d^**			
	TE1	Verification devices (optical counting, single pill ID, and barcode scanning)	
	TE2	Photos of reference pills in dispensing software	
	TE3	Summary table of the description of the product	
	TE4	Dispensing software only accepting 1 NDC to dispense	
**Organization^e^**			
	O1	Workload expectations	
	O2	Staff turnover	
	O3	Policies for communicating about errors	
	O4	Standards and policies for drug verification	
	O5	Types of organization (hospital, chain, or independent)	
**External environment^f^**			
	EE1	Similarities and size of pills	
	EE2	Wide variety of products available for similar active ingredients	
	EE3	Laws governing verification	
	EE4	Similar or different packaging	
	EE5	Limitation of reference database coverage	
	EE6	Drug supply and shortage	
**Examples of how different components interact and impact the performance of a system^g^**			
	TA-P4-TE1	A pharmacist who is comfortable using new technologies to verify medication filling from a technician or machine can benefit from various verification devices (optical counting, single pill ID, and barcode scanning).	
	TA-P2-O1-E2	When there are too many prescriptions to fill and high workload expectations, human vigilance and focus may decrease, leading to poor performance in medication verification.	
	TA-P5-O4-EE3	Obeying relevant laws and following policies that promote a safety culture and transparent communication of errors can enhance trust among co-workers and improve the efficiency of medication verification.	
	(TA1 and TA3 and TA4)-(P1 and P3)-E3-(EE1 and EE2)	Factors that impair human memorization and cognitive recognition activities (eg, small and similar pills, and distraction) can threaten the performance of tasks that require memorization and checking.	

^a^Tasks are actions performed in the medication verification process.

^b^Environment refers to work conditions that impact task performance.

^c^NDC: National Drug Code

^d^Technology and tools are devices and software used to assist in medication verification.

^e^Organization is about structural aspects that affect the process.

^f^External environment is about external factors influencing the system.

^g^Interaction examples within the SEIPS model, highlighting how various components interact and impact overall system performance in the medication verification context.

### Ideas of Human-Centered AI for Pharmacists and Prototype Feedback

In the last part of focus group 1 and the first part of focus group 2, pharmacists’ ideas of AI help and prototype feedback were collected. Using the HMT model concepts, we identified key factors contributing to the design of a human-centered AI for pharmacists performing medication verification tasks. These factors, as listed in Table 2, were identified through brainstorming and prototype feedback from participants.

Pharmacists indicated the use of a mental checklist during the medication process, emphasizing the need for the AI to check the same items (NDC, imprint, color, shape, size, quantity, and bottle label). As shown in Table 2, they stressed the need for transparency, requiring clarity on AI decision-making steps and rationale for rejections, and suggested stepwise checking indicators and predicted NDC probabilities for enhancing trust. For cognition augmentation, they highlighted automated alerts and pop-ups for error notification and problem-solving. Pharmacists wanted the AI to flag errors in real time and direct their attention accordingly. They also expressed a desire to understand AI’s predictive process and model performance, suggesting that bottle weight could assist with quantity verification. They found audio alerts with AI voice undesirable for design specifics. For effective coordination, pharmacists sought features such as the percentage match of the filled pill image with reference images and a calibrated understanding of user trust. They proposed a testing system with intentional errors for trust-building and addressing AI override concerns. The complexity introduced by confidence levels was noted as a potential decision-making influencer.

In focus group 2, pharmacists raised concerns about distinguishing white pills based on imprint and size through visual inspection alone. While they found the system’s use of red-green-blue values in pill image prediction helpful, they expressed the desire to physically handle the product. Concerns were raised about the top-down view of pills in the current prototype, which inhibits physical manipulation during internet-based verification. They suggested pill presentation on trays instead of bottles and voiced concerns about identifying mixed medications in 1 bottle. A heatmap of influential pixels and a low CI, when the model sees something unexpected, are helpful but do not completely alleviate the problem. The system’s prediction probability for each NDC was identified as a trust-building feature with suggested improvements, including the model’s second-closest result or a ranked probability of NDCs for medication images. Pharmacists appreciated the transparency of the proposed AI’s logical algorithm and error-reduction capabilities. They highlighted the model’s adaptability and suggested improvements such as zoomed imprints, reference images, and viewing pills from different angles. Pharmacists were also impressed by the model’s predictive capabilities and suggested improvements like more data sources and clear, nonconflict statements. Considering that prescription verification is time-consuming, they believed that AI could identify when human double-checks are necessary and when automatic prescription approval is safe. Confidence ratings provided by the AI system could make this possible with defined thresholds for human review. Subsequent focus groups further investigated these confidence ratings, leading to the design of pertinent elements.

**Table 2:** Mapping ideas, prototype features, and feedback to Human-Machine Teaming (HMT) model concepts. HTM categories include transparency (understanding the machine process), augmenting cognition (aiding human decision-making), and coordination (trust and teamwork between pharmacists and the artificial intelligence [AI] system). The research team prompted consideration of these principles while introducing the prototype AI system. Pharmacists offered comments on the prototype, deliberating its attributes within the HMT framework.

HMT category	Brainstormed ideas	Prototype feature and feedback	Exemplar quotes from FGs^a^
Transparency	Understand the steps of machine checking and assessing the same items as pharmacists: the NDC^b^, imprint, shape, size, quantity, and label on the bottle Understand the rejection from the machine and provide additional information to help pharmacists understand the issue	Discrepancy identification based on pixel input The probability of each predicted NDC Stepwise indicator of checking	“I was thinking more on the lines of would. You would have like a step, for example, here it’s going to look at the color, does the color match? Does the shape match? Does the identifier on each side match? They would check each step.” [FG1-a: observability, transparency] “I think I would want to see what it assessed. I don’t want to be guessing like, did it check the strength, or did it check the tablet size. I want to know if I did it or not. I’m not wondering if it was done.” [FG1-b: observability, transparency]
Augmenting cognition	Automated alerts and pop-ups to warn of mistakes and offer solutions Communicate errors or issues in real time Offer pop-ups and direct attention when the issue only matters If the pill did not match, the model tells the pharmacist what medication was filled in addition to what should be Confused pill by the machine Identify foreign objects, broken pills, or mismatched pills Ability to solve unexpected situations caused by changes in medication or sourcing Adapting to drug shortages and sourcing from nonstandard manufacturers with an incomprehensive database	Alert based on individualized CI threshold Low CI: unexpected input yields an uncertain prediction Pointing out key pill differences in hematocrit Improving human differentiation through zooming	“I guess an automated alert would be nice. I kinda you know where I was thinking the alert was that it actually won’t let it be since like wouldn’t be checked, I guess that are verified if it pops up that there’s something wrong with it which that can have issues in itself because something could happen. But that’s what I thought along the lines that it didn’t allow it to go through unless the image matched.” [FG1-a: directing attention, augmenting cognition] “If the medication fill is incorrect and the AI detects that, then I was hoping that they can tell me what medication was filled. It can pull images of all the medications and can tell us what was filled. I think we talked about how some of the mistakes and errors can be an opportunity for learning for the entire team, that’d be helpful.” [FG1-a: explore the solution space, augmenting cognition] “...there are changes that happen all the time, especially as you encounter shortages. Then we’re having to get products from not our standard manufacturers or getting them from abroad. I don’t know how frequently that happens in a community setting but sometimes with shortages, you get stuff from China, you get stuff from Europe. Those NDCs may not necessarily be in the database, that the system would have in place.” [FG1-b: adaptability, augmenting cognition]
Coordination	An accuracy, or how much percentage of the filled image matches the reference image User’s right to deactivate the system in case of errors Simple and calibrated understanding of trust for users A testing system with intentional errors to build trust Concerns around AI override Complexity could be added to decision-making by the confidence level	Calibrated trust through probability differentiation Improving trust with second source differentiation (eg, gravimetric data or pill count)	“I really liked the checkmark thing; I didn’t come up with that when P1 mentioned it. I liked that better. What I did was percent accuracy or percent my confidence or something. How they do, I don’t know, the facial recognition, it’s this percent of a match, so then I can figure out how confident the computer is in it, if that makes sense.” [FG1-a: calibrated trust, coordination] “The wrong med going out or me noticing that something’s wrong in there, then that would pretty much lose a lot of trust in it and so that would deactivate it. Just something going wrong.” [FG1-a: detectability, coordination] “...if there was a second source of data like gravimetric data, can I trust that the testing the weight of the tablet? So that would help confirm the identity of the drug but also the quantity. When the pharmacist is doing their check of the product, they’re checking the physical agent, but also the quantity. So, it would capture all those other elements of the pharmacist check as well.” [FG2-a: calibrated trust, coordination]
Design specifics	Implement visual verification with green checkmarks for matches of pill characteristics and red “X” for mismatches Confidence percent visualization Use color to indicate confidence levels Avoid pop-up overload to allow more focus on clinical work Avoid machine sounds for alert notifications	The probability of each predicted NDC Heatmap: black spot on the unexpected pills	“That’s my personal preference but I really hate listening to machine voices. If there’s going to be auditory sound, the alert, then I don’t want it to be a machine voice with something like the medication is wrong.” [FG1-a: design specifics] “In my mockup I said, do that at the very beginning and put a little green checkmark next to everything that I check. Like a little green checkmark next to the patient name does that matches the hard copy of the prescription, or if the drug doesn’t match, they put a little red X, so you can visually again see what it checked and what didn’t match.” [FG1-b: design specifics]

^a^FG: focus group.

^b^NDC: National Drug Code.

### Scenarios of Working with AI

**Figure 2:**
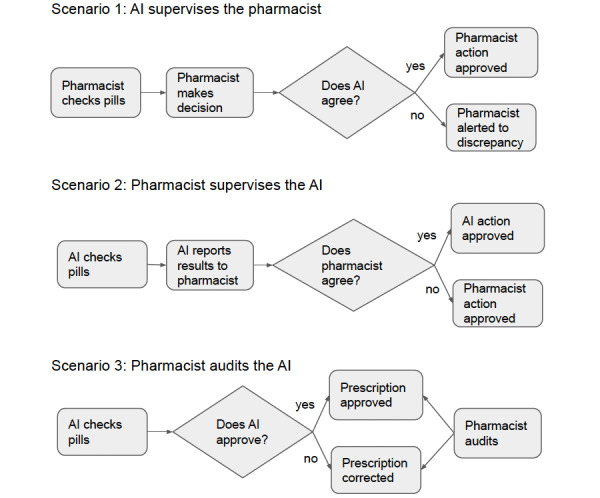
Scenarios for implementing human-centered AI. This figure presents 3 scenarios describing the roles of AI and pharmacists in the medication verification process. Scenario 1 depicts AI as a secondary checker, where the pharmacist’s decision is confirmed by the AI. Scenario 2 reverses the roles, showing the AI’s initial check with the pharmacist acting as the final check. In scenario 3, the AI primarily checks prescriptions, and pharmacists will audit the process and intervene as needed. AI: artificial intelligence.

Based on the insights from focus group 1, a total of 3 human-centered AI implementation scenarios were developed by the research team before focus group 2. In focus group 2, pharmacists examined the 3 scenarios for medication verification tasks (Figure 2), discussing their pros and cons in terms of workflow impact and patient safety.

In the first scenario, the AI acts as a vigilant guardian, double-checking during the medication verification process. Pharmacists recognized benefits such as increased accuracy, alert fatigue reduction, and enhanced peace of mind. They appreciated that the AI is not meant to act as a supervisor to the pharmacist but rather as a cooperative teammate, working in the background to identify the NDC. This AI system aligns with the pharmacist’s workflow by only alerting them when it identifies an NDC discrepancy, thereby functioning more as a guardian than a subordinate. However, concerns of complacency and AI overreliance added complexity, and no efficiency gain were raised. High AI accuracy, trust-building from experience, model understanding, and conflict-of-interest assurance were deemed necessary.

The second scenario involves the AI assessing pills before the pharmacist approves or disapproves them. Participants noted benefits like improved efficiency, streamlined workflow, and cost reduction. If the AI proved accurate, they could focus more on patient care. However, concerns about complacency surfaced, fearing less rigorous pharmacist double-checks if the AI already approved the pills. A threshold for AI accuracy was suggested for the first time, with additional checks like label consistency and transcription verification proposed. A hybrid scenario, blending an accuracy threshold and extra checks, was preferred for enhanced patient safety.

The third scenario involves the AI verifying prescriptions independently, consulting pharmacists only when its confidence is low. The identified benefits included freeing up pharmacists for other tasks and improving workflow efficiency. However, concerns about high-risk medications and significant errors surfaced. A hybrid approach was proposed, involving pharmacists in high-risk medication reviews and the AI in low-risk ones. Participants also proposed setting a threshold for the subset of prescriptions requiring pharmacist review based on the AI’s confidence level. The need for pharmacy-specific workflow adaptability, transparency with the Board of Pharmacy and the public, and an AI focus on prescription verification were highlighted. Some initial discomfort with not checking every prescription was expressed, but pharmacists recognized that this scenario might be the future of pharmacy. Nonetheless, several hurdles remain in implementing the third scenario. A considerable knowledge gap exists among pharmacists regarding the establishment of an accuracy threshold based on medication risk, and it remains unclear what consensus would be deemed an acceptable error rate for AI.

Overall, pharmacists recognized potential benefits across scenarios, such as increased accuracy, reduced alert fatigue, improved efficiency, and the ability to focus more on patient care. However, they raised concerns about complacency, AI overreliance, and error potential. Pharmacists generally favored a hybrid approach, incorporating an accuracy threshold, additional AI checks, and selective pharmacist involvement based on medication risk level and the AI’s confidence level.

### Refinement of the AI Prototype Interface

Pharmacist feedback provided valuable insights into the design preferences for a human-centric AI interface, emphasizing the need for simplicity and accessibility. Preferences were articulated for specific design features, such as juxtaposing pill images with their packaging for quick comparisons. Additionally, they favored displaying only essential information, including NDC rank and AI accuracy, to prevent overwhelming users with excessive data. Pharmacists also found value in the accept, reject, or unsure options and the inclusion of details about drugs the AI model frequently confused with the target drug. Despite concerns from some pharmacists that numerical values could lead to practice variations due to individual interpretation, others recognized the value of knowing the predicted accuracy for the NDC, especially when a defined threshold for auto-approval was lacking. They proposed a balance between numerical values and visual representations, such as confidence graphs, supplemented by tutorials or definitions for a clear understanding of the data.

**Figure 3:**
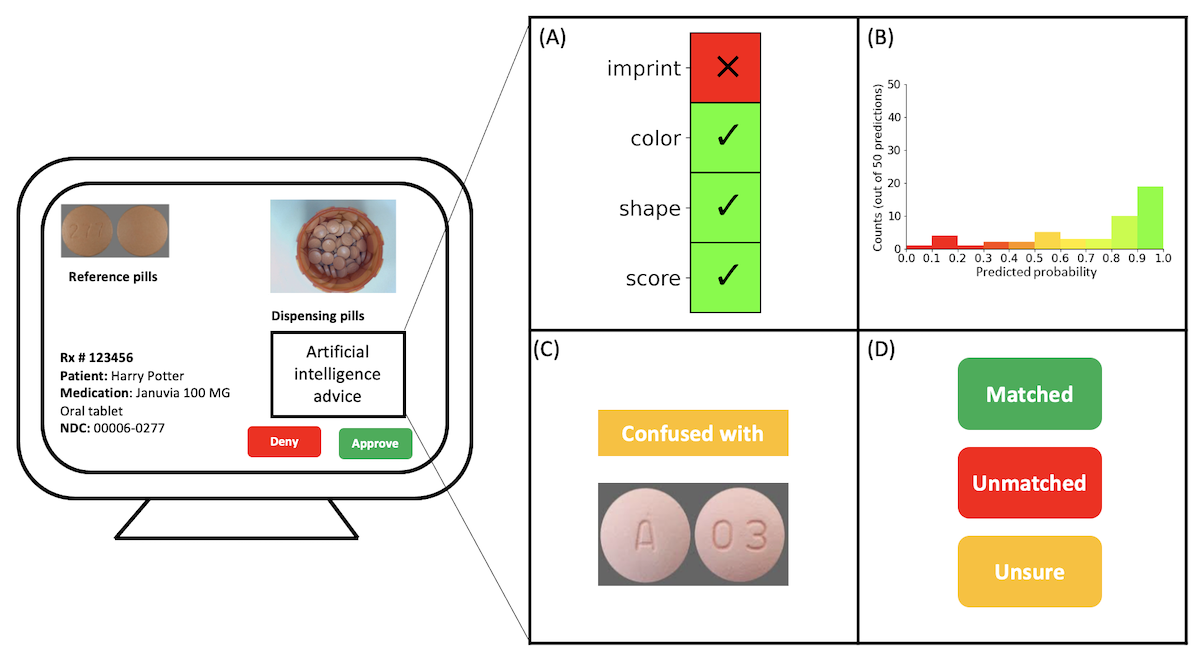
Prototype visualization for a human-centered, AI-assisted dispensing interface. (A) Comparison of pill characteristics from the National Library of Medicine Pillbox data set between the AI-predicted NDC and the expected NDC on the prescription. Differences are denoted by a red “X,” while identical characteristics receive green checks. (B) A histogram of the predicted probabilities for the AI-predicted NDC, where a flat distribution indicates low certainty and a peak suggests high certainty. (C) The AI-provided “confused” pill based on the second-highest AI-predicted NDC. (D) NDC match status: green for a match, red (unmatched) for nonmatches, and yellow (unsure) if AI predictions are below a certain threshold. AI: artificial intelligence; NDC: National Drug Code.

In response to pharmacist preferences, a comprehensive interface (Figure 3) was engineered, integrating elements derived from pharmacist insights to augment the interpretability of our deep learning model’s recommendations. In alignment with the pharmacists’ mental checklist, we introduced checkmarks to compare pill characteristics—sourced from the National Library of Medicine’s Pillbox data set—between the AI-predicted NDC and the prescription’s expected NDC. To represent the AI’s confidence level, we used a histogram showing predicted probabilities for the AI-identified NDC. For the pharmacists to better understand and trust the AI’s recommendations, we indicated the pills the AI might confuse based on the second most probable AI-predicted NDC. To enhance the AI system’s directive capabilities, we incorporated an NDC match status feature, adding more guidance to pharmacists.

## Discussion

### Overview

In this study, we collaborated with pharmacists to develop a human-centered AI interface designed to aid in error prevention during medication dispensing. Our investigation focused on identifying key features in current medication verification processes to understand where an AI model could be beneficial. Pharmacists actively participated in brainstorming features for the AI support system, particularly concerning usability, interpretability, and trustworthiness. They also provided feedback on our early prototypes based on the principles of HMT. We gained insights into pharmacists’ preferred work styles with AI and subsequently designed a dispensing interface equipped with human-centered AI features to enhance human-machine collaboration and prevent dispensing errors.

AI, particularly the recent breakthroughs in Generative Pre-trained Transformer 4, is revolutionizing various industries, including health care [31-33]. Meanwhile, machine learning and deep learning have demonstrated significant potential for enhancing health care quality and safety. Early disease diagnosis through medical imaging serves as an example of these technologies’ maturity [13]. However, despite their outstanding performance metrics, the deployment of AI technologies in clinical practice remains sparse. This can be partly attributed to the “black box” nature of deep learning AI systems, which engenders low trust in critical fields like health care. Trust in technology was improved due to the accelerated adoption of telemedicine and teleprescribing practices during the SARS-CoV-2 pandemic, which demonstrated the practicality and reliability of these technologies in critical health care contexts [34,35]. The pandemic’s unique challenges led to a wider acceptance and trust among both health care providers and patients in technology-driven health care solutions, underscoring the importance of technological adaptability in times of crisis [36]. Some researchers advocate for interpretable AI in health care, arguing that elucidating AI’s working mechanisms and presenting information through suitable interfaces can foster trust, augment the transparency of AI-driven decision-making, mitigate bias, and enhance human-machine teaming performance [37-40]. However, other researchers caution against making interpretability a prerequisite for AI models in the absence of appropriate explainability methods. They advocate for rigorous internal and external validation processes as a more direct path to trustworthy AI systems [41]. A significant barrier to effective, explainable AI in health care is that solutions often derive from developers’ intuition rather than users’ experiences and capacities to use AI outputs [41,42]. For example, common post hoc explainability methods, such as saliency maps to highlight image areas influencing AI decisions, can be unreliable and confusing for the user, lacking actionable information [43].

To address this, our study leveraged human-understandable features—a checklist of pill characteristics mirroring the cognitive process of pharmacists during dispensing verification tasks. Originating from the pharmacist’s own mental schema, this intuitive feature can engender trust and may contribute to better system understandability. We aim to improve AI system adoption while also reducing overreliance on it. To accomplish this, we introduced a graphical representation of uncertainty (a histogram) along with 3 options for users to choose from: accept, reject, or unsure. Specifically, these features help pharmacists understand the confidence level of the model’s advice. By giving a clear visual and choices, pharmacists can easily assess whether the model is sure about its recommendation. This also empowers them to make an informed decision about whether to reclaim the authority of approving or rejecting the dispensing in a timely manner. Our human-centered AI design process commenced with an in-depth understanding of task scenarios, constraints, and end users’ perspectives using the SEIPS model. Future work should be conducted to rigorously test and evaluate the use of such AI systems in real-world practice, with an emphasis on their evaluation of the performance of teaming with humans.

Despite the proven efficacy of AI systems in health care, concerns remain among professionals about potential encroachment on their roles, often perceived as challenges to their professional autonomy. This sentiment is amplified by extensive research investigating AI’s capacity to replace tasks undertaken by health care professionals [23]. In contrast, the “teaming” concept between AI and humans has been proposed, where each entity leverages the other’s strengths to enhance outcomes. As revealed by Henry et al [22], health care professionals typically do not perceive AI as a replacement tool. Instead, they envision it as a collaborative partner, steering their attention toward patients or situations requiring immediate intervention. This corresponds with this study’s feedback, where pharmacists desired to be notified of any anomalies. Therefore, our system design focuses more on supporting rather than replacing the dispensing verification processes conducted by pharmacists.

In terms of teaming styles and AI automation level, we explored 3 modes of integrating AI and pharmacists. Pharmacists expressed a preference for adaptable teaming styles (a mixture of scenarios 2 and 3 from Figure 2), dependent on the varying risk levels associated with different medications. For instance, in cases of low-risk medications, pharmacists were comfortable with the machine conducting medication verification while they assumed supervisory roles. Conversely, in situations involving high-risk medications, it was crucial for pharmacists to maintain situational awareness and grasp tasks exceeding the capabilities of machine intelligence. In such high-risk scenarios, the AI system needed to provide transparency to signal the necessity for human intervention and facilitate issue resolution. This resonates with the ideas of Saenz et al [44], who noted that different decision-making scenarios, varying in risk-level consequences, necessitate differing capacities for human-machine teaming. It also echoed the study from Parasuraman et al [45], discussing “adaptive automation,” which refers to the ability of a system to change its automation levels based on contextual factors, user needs, or system states. Designers and engineers should consider evolving and flexible approaches to integrating automation into human-machine teaming. Our findings suggested insights into setting teaming styles based on medication risk, local regulatory requirements, and individual pharmacists’ error tolerance levels. High-risk medications necessitated a balance of authority leaning toward humans and increased AI transparency to enhance situational awareness and solution exploration. However, pharmacists struggled to agree on how to quantify incorrect medication dispensation risk or establish the optimal threshold for human intervention during verification. Future research may investigate how to determine these medication risks and associated thresholds for human intervention to prevent overtrust.

While the findings of this study provide significant insights, several limitations warrant consideration. The study’s sample comes from purposive sampling, composed of 8 pharmacists exclusively from 2 networks in Minnesota and Michigan, and may not represent the entire range of experiences and perspectives across different geographical regions. Second, while this study effectively gathered pharmacists’ feedback on iterative prototypes—a key aspect of human-centered AI development—it lacked contributions from other stakeholders such as patients and administrative or regulatory staff. Their input could bring diverse perspectives to bolster safety measures. Although these approaches provide important initial design insights, they are no substitute for comprehensive real-world testing. Our reliance on web-based focus groups is another limitation. These groups offer insights into pharmacists’ perceptions of AI but do not capture the hands-on context needed for dispensing verification system design or usability assessment. Observing pharmacists in their work environment would have provided more actionable insights. Hence, the findings should be viewed as suggestive, representing perceptions rather than actual behaviors. We believe it is crucial to test the AI system in simulated or real-world settings for a true measure of its performance. Implementing these empirical data in subsequent prototypes will enhance the system’s applicability in real-world pharmacy practice, thereby optimizing its effectiveness in teaming with pharmacists to minimize dispensing errors.

### Conclusion

This study underscored the importance of designing human-centered features and enhancing the interpretability of AI systems through the incorporation of measures like understandable process illustration, confidence visualization in AI advice, and supplementary information to enhance situation awareness. Our findings explored the human-machine teaming style, underscoring the importance of creating user-centered strategies to augment the application of AI systems and establish reliable and trustworthy teaming with health care professionals.

## Appendix

Multimedia Appendix 1 Supplementary interview guide 1.

Multimedia Appendix 2 Supplementary interview guide 2.

Multimedia Appendix 3 Supplementary interview guide 3.

## Abbreviations

AI: artificial intelligence
HMT: Human-Machine Team
IRB: institutional review board
NDC: National Drug Code
SEIPS: Systems Engineering Initiative for Patient Safety

## Data Availability

Data sharing is not applicable to this article as no data sets were generated or analyzed during this study.
