# Supplementary material for: Designing Human-Centered AI to Prevent Medication Dispensing Errors: Focus Group Study With Pharmacists
Source: JMIR Form Res. 2023 Dec 25;7:e51921. doi: 10.2196/51921 (PMC10775023; doi:10.2196/51921)
Supplement: Multimedia Appendix 1 [file formative_v7i1e51921_app1.docx]

Human-Machine Teaming Interview Guide

Adapted from 2018 The MITRE Corporation

NIH NLM Images study - Wave 1

University of Michigan

Fall 2021

**--Focus group #1 [2 hours]--**

# Introductory Material [45 minutes]

## Introduction [10 minutes - 530pm]

Thank you for joining us and agreeing to participate in this study investigating artificial intelligence, human-decision making, and pharmacy practice. In this phase of the project, we will ask you to participate in design sessions, in which we ask you to complete interactive design activities, respond to questions, and have you participate in writing activities to help stimulate our discussion. Over the next 3 sessions, this will help us understand your current work, brainstorm ideas for new tools, and evaluate how those tools might work. The goal of this study is to develop a novel technology designed to safely and effectively support pharmacy staff in their work of dispensing prescription medications correctly to patients. As a reminder, this Zoom call will be recorded. If you have any questions before we begin, please feel free to ask them now. [pause] Thanks, I will hit the record button now.

[introduce study team members] - photo, name, title, role on project

This is intended to be a safe space, in which you can talk freely and openly about the topic at hand. There are no right or wrong answers. Given the group setting, we cannot guarantee anonymity of your participation, however, we ask all participants to keep the contents of the discussion confidential and not reveal the identity of your fellow participants to others.

To begin, let’s start with a simple ice breaker. This will allow us to get to know each other and hopefully feel a little more comfortable talking with each other. Let’s now go around to each person and answer the following questions (Corey to start as an example):

1. What is your name?
2. Where do you currently practice?
3. How many years of experience as a practicing pharmacist?
4. Do you currently use artificial intelligence in your pharmacy workflow?
5. What is a hidden talent?

## Paint Picture of Envisioned Autonomy [5 minutes - 540pm]

Imagine that you are in your role at the pharmacy dispensing medications. You’ve been notified that a filled prescription is ready for your review and you pull the order up in the dispensing software. The prescription has already gone through a pre-verification in which things like drug interactions have been verified. During this final step in the dispensing process, you are to make sure that the correct medication is in the medication bottle which was filled by another team member in the pharmacy.
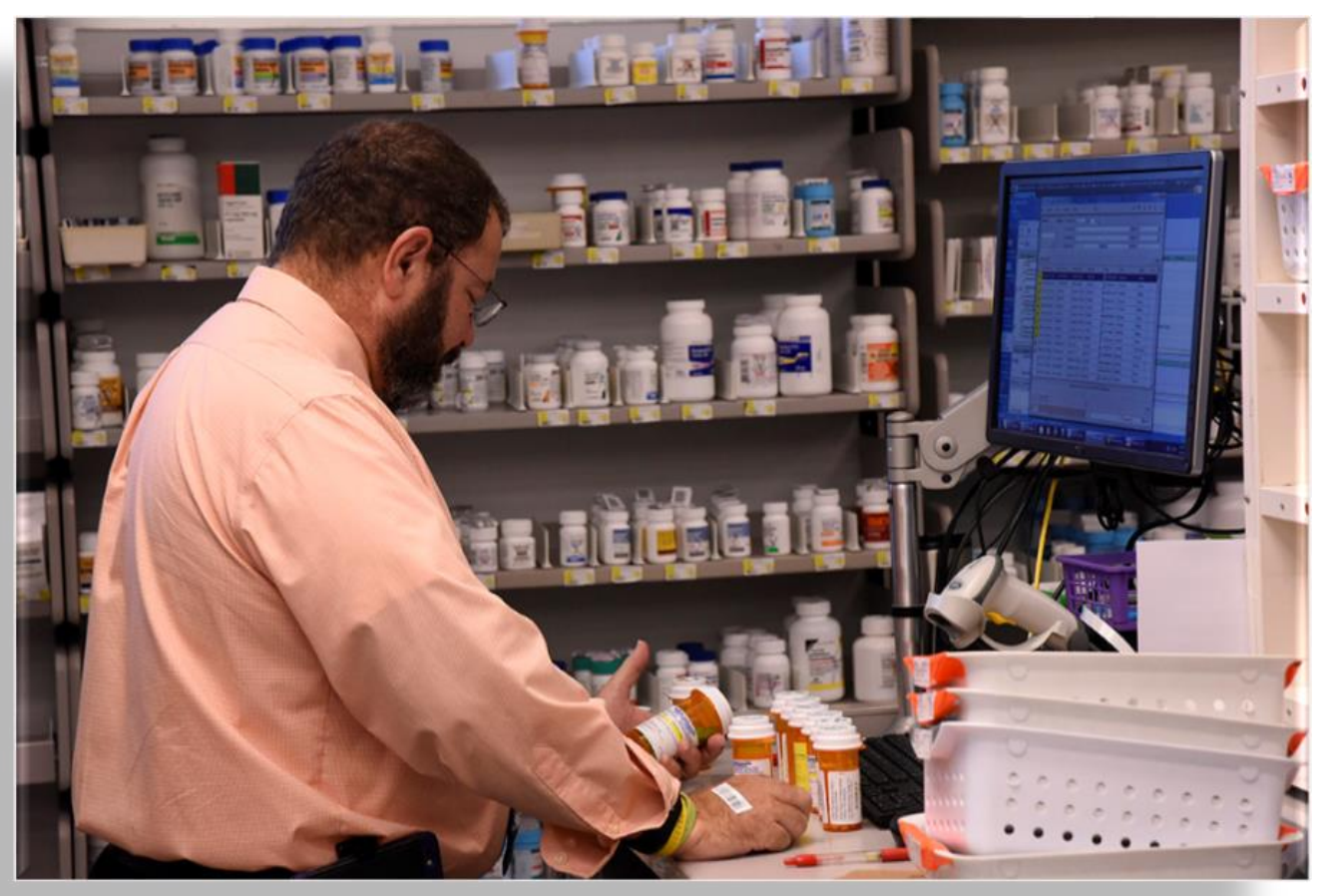


Despite the use of technologies, such as bar code scanning in the pharmacy, you’ve encountered errors in the past and you want to make sure that the prescription bottle contains the correct medication. The pharmacy is busy and you have many prescriptions ready to be verified, so you are feeling some time pressure to get through all of these prescriptions before your shift ends.

The questions that follow will help us to first understand how you currently perform these verification tasks and how you respond to incorrectly filled prescriptions.

## Top Challenges [10 minutes - 540pm]

1. What are the primary tasks you’re responsible for when verifying the correct medication was filled?
   1. What are the steps in your process?
   2. What do you look for when verifying the contents of prescription bottles?
   3. Which of these steps are the most difficult cognitively?
      1. What makes them difficult?
      2. What makes them easier?

## Optional: Critical Decision Method Probe [15 minutes - 550pm]

1. Can you think of a particularly challenging time when you were verifying the medication dispensed?
   1. Describe what made the process challenging
   2. What were the circumstances surrounding this time?
   3. What were your primary concerns during this time?
2. Can you think of a time when you discovered an incorrectly filled medication?
   1. How did you identify this?
   2. What tipped you off that it was filled incorrectly?
   3. How did you go about resolving this issue?
3. Challenges of remote verification [poll the participants]:
   1. For those that **currently/have experience** using remote verification: what are the challenges with using pictures instead of the actual bottle?
   2. For those **not currently/without experience** using remote verification: what do you think would be the challenge of using pictures instead of the physical bottle?
   3. What are the advantages and disadvantages of remote verification?

This concludes the questions we have about your current work process of verifying medications. Are there any other thoughts you’d like to share before we move on to the next activity?

## Paperclip Activity [5 minutes - 605pm]

Paper clip task:

Now we’re going to do a warmup brainstorming activity. The purpose of this activity is to help jumpstart the brainstorming process as well as get everyone familiar with using Google Slides. We’ll be using Google Slides for a larger activity in the second half of the session. Brigid just sent each of you a link to your own personal Google Slide via chat. Please click on the link now. I’ll give everyone a moment to open their document.

Was everyone able to open the file? [wait for responses - troubleshoot if needed]

[Briefly demo Google Slide (slide 1) - show the text box, shapes/arrows, and line/scribble tools]

Now click on slide 2 on your left. Previous research has shown that performing a warm-up brainstorming activity helps people generate more ideas throughout a brainstorming session. When I tell you to start, use your Google Slide to write down or draw out as many uses of a paperclip that you can think of in 1 minute. The more creative the ideas the better. Are there any questions? OK, time starts.. now. Please start writing down your ideas. [Wait 1 minute]

Alright time’s up. Now I’d like to go around and ask each person to share their favorite examples [jot down the examples people came up with on your own slide slide]

Wow - those are some really creative ideas.

Great, this concludes the first section of our meeting today. Let’s take a 2-2.5 minute break and regroup at __.

**[2 minute break]**

# HMT Knowledge Audit : DESIGN SESSION #1 [60 minutes - 615pm]

## Initial Design Activity[10 minutes]

Welcome back! Let’s think back to the scenario I described previously and I’ll expand on it a bit more.

Imagine that in this pharmacy, you verify these prescriptions remotely, that is, you don’t actually handle the physical bottle to inspect the pills inside the prescription bottle, rather, you have a photo of the pills that were taken by one of your team members in the pharmacy. A screenshot of a typical remote medication verification screen is shown on the slide. Our team has been building a computer model that can identify the medication product using the picture of the pills inside the medication bottle.

Our goal in this session is to help us envision an artificial intelligence tool that can be employed to help you in the verification process to avoid dispensing errors and make you more efficient with your time. To achieve this goal, we have recruited you to act as advisors and assist with brainstorming solutions and prototyping designs that could safely and effectively support your work in the pharmacy.

We’ll start by brainstorming ways in which an artificial intelligence tool might be designed to support you in this task. Go back to your Google Slide deck, and click on the 3rd slide. In this activity we would like you to imagine that you could design a computerized tool to automatically assist with the verification process. On this slide, please take 10 minutes to start mocking up the slide to demonstrate how you might design artificial intelligence to help support you in your work verifying medications at the pharmacy. Here are some questions to think about as you do this task:

1. What would that automated assistance look like?
   1. When would it communicate with you?
2. How would this AI work?
   1. What would the AI tell you?
3. What features would you like to see?
4. How would the AI alert you about an incorrectly filled medication?

Don’t worry about finishing as we’ll come back to the slide after we go through some questions to further stimulate your thinking.

**[10 minutes for activity]**

Now we want to ask you a series of questions that are aimed at identifying important characteristics of the artificial intelligence and then we’ll end by asking you to return to your mockups to contribute any additional ideas for how the artificial intelligence might communicate with you about the work it is performing.

## Past and Future: Predictability, Exploring the Solution Space [5 minutes - 625pm]

1. When verifying medications inside prescription bottles, how predictable or variable is the task?
   1. What information about the medication is critical to have?
2. If you could have an automated alert that stood watch for you, is that a need?
   1. What would you want it to tell you? (e.g., an alert notifies you when the medication inside the bottle is a discrepancy from the medication recording in your dispensing software)

## Big Picture: Observability [10 minutes - 630pm]

1. What’s the overall decision timeline of verifying medication? (e.g., how quickly do you need to make a decision? how does your information needs change as you are verifying the medication dispensed?)
2. If you had artificial intelligence to support you in this task, what would you want the automation to tell you as it’s working for you?
   1. When should it communicate to you?
   2. How should it communicate to you?
3. Can you describe a scenario where you might be confused about what the AI could be doing? (e.g., how the dispensing software provided a recommendation regarding the medication to be dispensed)
4. What are the key vital signs to know that you are making correct decisions?
   1. eg, color, shape, size of pills
   2. The machine might be looking at these features, do you think there are things the AI could miss?
5. In what ways might the AI help you coordinate more effectively, if at all?

## Anomalies: Calibrated Trust, Directing Attention, Adaptability [10 minutes - 640pm]

1. What are the biggest anomalies you worry about?
   1. Are there particular pill mismatches that could cause significant harm to patients?
2. What are medication mismatches/confusions that you are particularly sensitive to?
   1. How might the AI be aware of these?
3. What aspects of this artificial intelligence might help you determine how much and when to trust it?
4. Are there nuances that people pick up over time to know things are not right? Can you describe an instance when you spotted a deviation from the norm, or knew something was amiss?
   1. How might the AI notify you of or anticipate this?
5. Can you think of certain conditions in which artificial intelligence might provide unreliable information? (e.g., the reference image contains discrepant features of a pill for the same medication - the manufacturer changed the physical characteristics of the pills without update to reference image)
6. Can you think of a time that you needed to improvise while verifying medications?
   1. Are there things this artificial intelligence could do to help you adapt? (e.g., when you didn’t have a reference image? or you couldn’t tell if the right medication was inside the bottle)

**[2 minute break]**

## Noticing: Directing Attention, Information Presentation [5 minutes - 650pm]

1. Can you think of a time when you missed something important that didn’t pop out at you in a clear way while verifying medication?
   1. How might AI have helped you notice what you missed?
2. Can you imagine a role for artificial intelligence to direct you to see what you’re looking at, to get a common frame of reference?
3. Have there been times that you wish you were notified of a mismatch between the medication inside the bottle and the medication to be dispensed?
   1. Would that have made a difference in your decision making?
   2. What would that notification look like?
      1. What information would it contain?

## Self-monitoring: Common Ground, Calibrated Trust [10 minutes - 655pm]

1. Can you think of a time when you knew you were task saturated/overloaded and had to ask for help?
2. If a new person on your team were to take over your job of verifying medications, what would you be most concerned about?
   1. What part of this task would you feel most uneasy about if a brand-new person was doing it?
3. What indicators might you use to know that something’s amiss with your automated partner’s performance, or its intent or assumptions? (e.g. the situation changed, and the system’s algorithms aren’t calibrated, or applicable)

## Improvising: Adaptability, Directability [5 minutes - 705pm]

1. What are some example situations in which you have had to rapidly improvise a plan? (e.g., either to handle a threat or take advantage of an opportunity)
   1. Using this example, how might your automated partner and you take advantage of this opportunity/handle this issue?
2. When the situation has changed, what might you need to understand and to direct the artificial intelligence teammate’s response to this change?

## Job Smarts [10 minutes - 710pm]

1. If the pharmacy was giving you an artificial intelligence/digital team member (like decision-making algorithm/software), what are the top things you’d like it to do for you?
2. On the flip side, what would you not want your AI/digital team member to do?
3. If you had automation to help, what expert knowledge would it need to be aware of to be effective?
4. Are there administrative, information seeking, or representation tasks that your automated partner could do to make the team more efficient and effective? (e.g., rapid lookup of confounding medications that are commonly mistaken for each other)

## Envisioned Model [8 minutes + 10 minutes for sharing - 728pm]

Thanks for your active participation discussing these questions. As I mentioned previously, now we’re going to revisit your mockups from the AI medication verification brainstorming activity.

1. In this activity we would like you to imagine that you could design a computerized tool to automatically assist with the verification process. What would that automated assistance look like?
   1. What features would you like to see?
      1. When would it communicate with you?
   2. How would this AI work?
      1. What would the AI tell you?
   3. How would this information be communicated to you?
   4. How would the AI alert you to an incorrectly filled medication?

Going back to the google drive slide you worked on earlier, please take some more time to improve your ideas and revise them based on our discussion with each other. We want you to tell us the ways in which you would want artificial intelligence to interact with you during this task. Then we will reconvene as a group to share what each of you came up with.


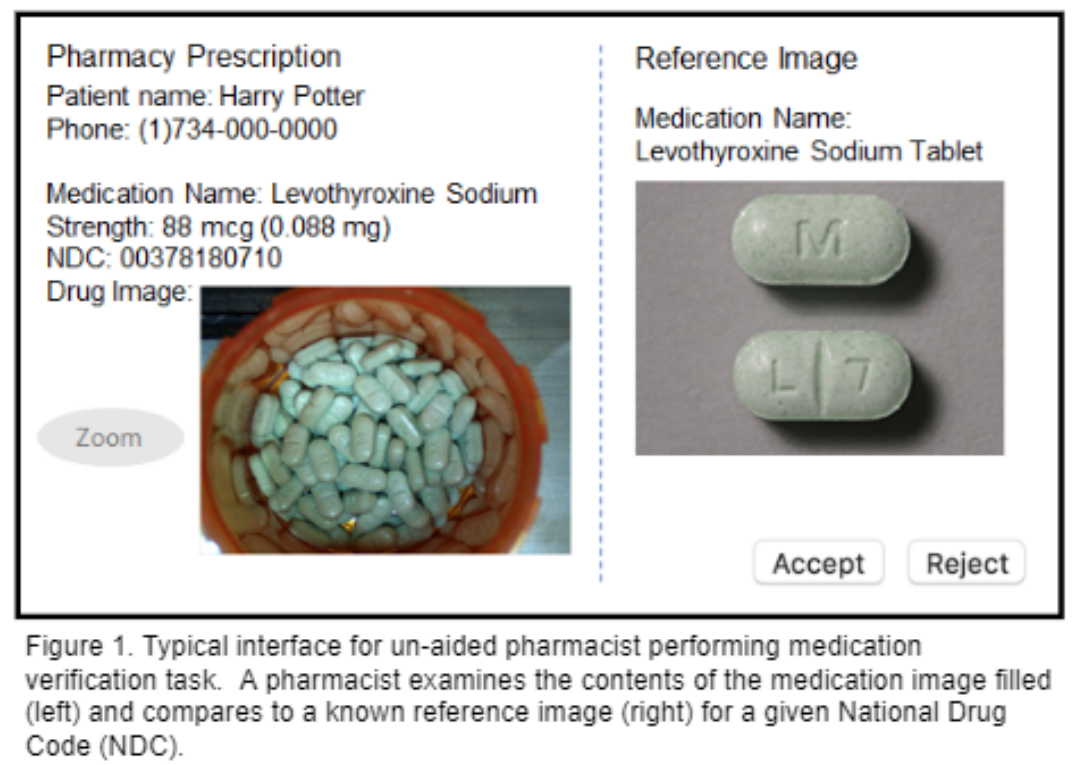


[reconvene the group - and ask them to share their screen and give a short synopsis of their ideas - 1 minute each]

*Facilitator note:* Ask questions related to the concrete ideas the group comes up with.

# Closing [2 minutes]

What a great session this was! As a reminder this was the 1st of 3 design sessions. Our next steps are to synthesize what we’ve learned from you today and incorporate into the design of our model to verify the contents of medication bottles for specific oral prescription products. Over the next 12-16 weeks, we will work on implementing your ideas. At our next session, we will show you some of the ways we’ve incorporated your input into our model and ask you to create some more as well as provide your honest feedback and critique. We want to design the best possible tool and you won’t hurt our feelings if there are things that you don’t like or think are useless.
