# Supplementary material for: Designing Human-Centered AI to Prevent Medication Dispensing Errors: Focus Group Study With Pharmacists
Source: JMIR Form Res. 2023 Dec 25;7:e51921. doi: 10.2196/51921 (PMC10775023; doi:10.2196/51921)
Supplement: Multimedia Appendix 2 [file formative_v7i1e51921_app2.docx]

NIH NLM Images study - Wave 1, Session 2

University of Michigan

Winter 2022

**--Focus group #2 [2 hours]--**

# Introduction/Welcome

## Introduction [10 minutes]

Welcome back to the second focus group for our study, AI in the Pharmacy. Thank you for taking the time to meet with us again. You may notice some new faces in today’s group. During each round of focus groups, you may be in sessions with members you haven’t seen before. To start today’s session, I’d like to briefly review the purpose of these focus groups and the ground rules. After that we’ll do a quick round of introductions.

The goal of this study is to develop a novel technology designed to safely and effectively support pharmacy staff in their work of dispensing prescription medications correctly to patients. In this phase of the project, we are asking you to participate in the design sessions. In these sessions, you will complete interactive design activities, respond to questions, and participate in writing activities to help stimulate our discussion. This will help us understand your current work, brainstorm ideas for new tools, and evaluate how those tools might work.

This is intended to be a safe space in which you can talk freely and openly about the topic at hand. There are no right or wrong answers. Given the group setting, we cannot guarantee anonymity of your participation, however, we ask all participants to keep the contents of the discussion confidential and not reveal the identity of your fellow participants to others. As a reminder, this Zoom call will be recorded. If you have any questions before we begin, please feel free to ask them now. [pause] Thanks, I will hit the record button now.

# Cost-sensitive modeling activity #1

Now we’re going to talk about dispensing errors. Not all dispensing errors are created equal - they vary in severity. Some errors are unlikely to cause harm to the patient whereas other errors could result in hospitalization or death. For example, dispensing X instead of Y is unlikely to harm a patient whereas incorrectly dispensing A instead of B could result in [name the severe outcome].

In our next activity we’re going to show you pairs of drugs. You will see the drug that was prescribed and the drug that was dispensed. On a scale of 0 to 3, we’d like you to rate the severity of the error. 0 indicates no harm is likely to happen to the patient. 3 indicates severe harm is likely (life-threatening, hospitalization, or disability).

Link to Qualtrics survey:

[Give participants approximately 5 minutes to complete the survey.]

Thanks, everyone, for completing the survey. We’ll revisit it again at the end of today’s session.

# Icebreaker for Discussion

Let’s do a quick round of introductions by having everyone answer the following questions (Corey to start as an example):

- What is your name?
- Where do you currently practice?
- What did you want to be when you grew up?

# Transparency

During the first focus group we talked quite a bit about what you would like the model to show you. The feedback we received is you would like to know what the model is checking to make its assessment. We heard you say you would like the AI to check pill features such as the NDC, imprint, color, shape, and size as well as more global features like the quantity of pills in the vial, the label on vial, and all pills/objects in vial. You also wanted to know how the AI makes its predictions and how well the model performs.

Having this understanding of how the model works and makes its predictions is called transparency. Today we’re going to focus our discussions on transparency of the model. Transparency is made up of two major components - observability and predictability. Let’s start by defining observability and transparency:

Observability means the system proactively communicates with you to let you know what it’s thinking and doing, and tells you how far along it is in accomplishing your joint work. For example: your GPS/directions app telling you that it’s rerouting you because there is an accident ahead.

Predictability means the system communicates with you about its intentions, goals, and future actions in various contexts. Example: The green "armed" light on an auto-pilot control panel informs the pilot that when the approach glide path starts, a descent will automatically begin.

I would like you to keep the idea of transparency in mind during the presentation of our AI model. After the presentation, we’ll have a group discussion that focuses on the transparency of our model.

# Model Presentation - Raed Al Kontar and Qiyuan Chen

Raed Al Kontar is going to lead the discussion about our AI model. Raed is one of the co-investigators on the study and is an assistant professor in the Industrial & Operations Engineering Department here at the University of Michigan. Raed leads a data science focused lab and his team are developing the AI model for this study. Today he is going to talk to you about the AI model being created and show you a mock-up. After his presentation, we’ll have a group discussion to get your feedback on the model. Please feel free to ask use questions throughout the presentation

1. Presentation by Raed and Qiyuan
   1. Describe the model and its abilities/performance
      1. Components of the check
      2. Description of the development process
   2. Show mock-up

## Follow-up Discussion

Thanks, Raed and Qiyuan. Thinking about their presentation and the concept of transparency, I would like to have a discussion about the model. As a reminder, transparency is made up of observability and predictability. We’ll start with the observability component. Observability is how the system proactively communicates with you to let you know what it’s thinking and doing, and tells you how far along it is in accomplishing your joint work. For example: your GPS/directions app telling you that it’s rerouting you because there is an accident ahead.

1. To what extent is the model observable?
   1. How does the model’s output help you verify medication?
2. What aspects of the model should have greater observability?

Great. Now let’s shift gears and discuss the model’s predictability. Predictability is how the system communicates with you about its intentions, goals, and future actions in various contexts.

1. What aspects of the system offer good predictability?
2. What are aspects of the model that need greater predictability?

Additional comments regarding:

1. Workload
2. Trust
3. Perceived safety

[2-5 minute break]

# Augmenting Cognition

Welcome back! I’m now going to lead the next part of today’s session. Jessie Yang, an Assistant Professor in the Department of Industrial and Operations Engineering, will be helping out with this discussion and she is one of the co-investigators on this study. Her research is focused on the interactions between human and automation and human and robots. Today we’ll talk to you about different approaches AI can support pharmacists in the workplace that you all identified in our first focus group. We’ll review each of the 3 scenarios one by one and get your feedback on each. Then we will open up for some ad ditional comparison between the 3 approaches.

## Scenario 1: AI supervises the pharmacist

In scenario #1, the AI supervises the pharmacist.


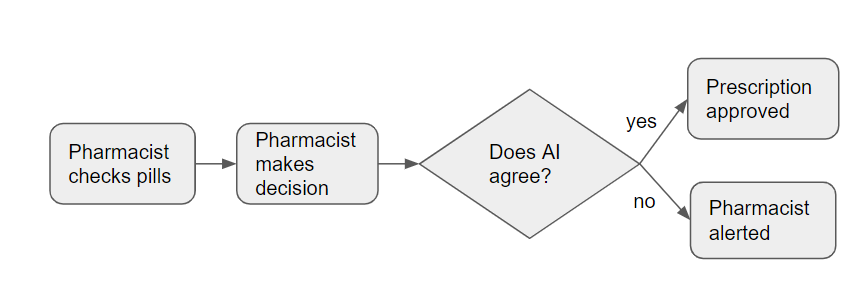


The pharmacist checks the pills and decides whether the dispensed drug matches the prescribed drug. The AI then reviews the decision. If the AI agrees, the prescription is approved. If it does not agree, the pharmacist is alerted that there is a possible mismatch.

**DISCUSSION**

What are the potential benefits of this approach?

- Workload
- Safety

What are the potential risks of this approach?

Hypothetically speaking, if the aid is not 100% accurate, what’s your tolerance for risk and perspective on benefit?

If we could change the procedure, what changes would you like to see to make it more trustworthy?

To what extent would you trust this system?

- What aspects would make you trust it more?
- What aspects would make you trust it less?

## Scenario 2: AI supervises the pharmacist

In scenario #2, the AI supervises the pharmacist. This is similar to the first scenario, but the roles of the pharmacist and AI are switched.


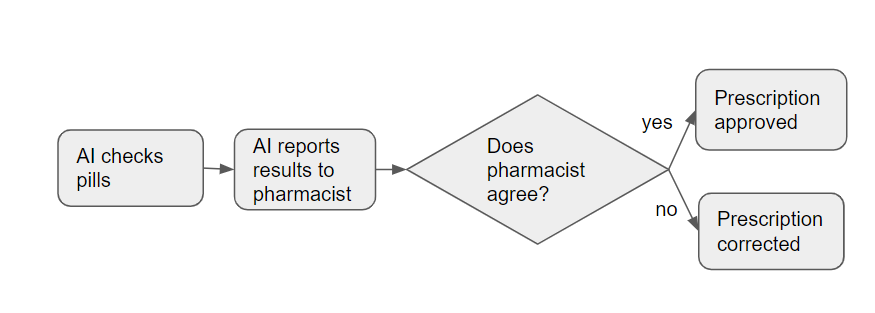


Here the AI is responsible for the first check. The AI reports to the pharmacist whether the dispensed pills are a match for the prescribed drug. If the pharmacist agrees that the prescription is a match, the prescription is approved. If the AI and/or the pharmacist find a discrepancy, the prescription is corrected.

**DISCUSSION**

What are the potential benefits of this approach?

- Workload
- Safety

What are the potential risks of this approach?

Hypothetically speaking, if the aid is not 100% accurate, what’s your tolerance for risk and perspective on benefit?

If we could change the procedure, what changes would you like to see to make it more trustworthy?

To what extent would you trust this system?

- What aspects would make you trust it more?
- What aspects would make you trust it less?

## Scenario 3: AI acts alone

In the final scenario, the AI acts alone. The pharmacist is largely removed from the process in this scenario with their role being limited to auditing the AI’s performance or being called upon by the AI to help when the AI is unsure.


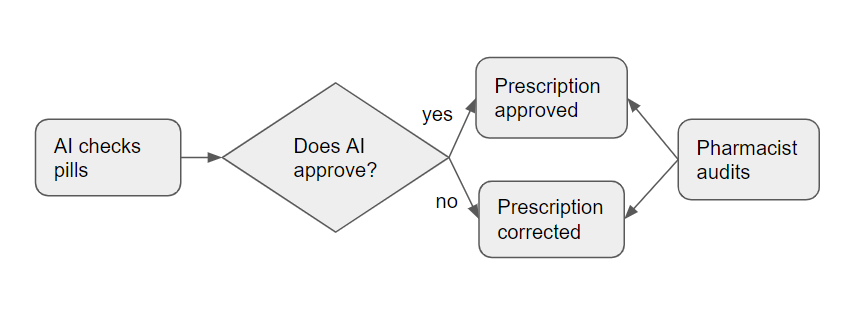


**DISCUSSION**

What are the potential benefits of this approach?

- Workload
- Safety

What are the potential risks of this approach?

Hypothetically speaking, if the aid is not 100% accurate, what’s your tolerance for risk and perspective on benefit?

If we could change the procedure, what changes would you like to see to make it more trustworthy?

To what extent would you trust this system?

- What aspects would make you trust it more?
- What aspects would make you trust it less?

## Member Discussion

1. Pros/cons/preferences for the 3 scenarios.
2. Critique of the 3 scenarios

| **Scenario 1**: AI supervises Human | | **Scenario 2**: Human supervises AI | | **Scenario 3**: AI acts alone | |
| --- | --- | --- | --- | --- | --- |
| Pro | Con | Pro | Con | Pro | Con |
|  |  |  |  |  |  |
| Order of preference? | | | | | |
|  | |  | |  | |

# Cost-sensitive modeling activity #2

That was a great discussion! For today’s final activity, we’re going to revisit the survey you completed at the beginning of the session. While we’ve been talking, one of Raed’s students has been updating the model based on the severity scores you provided. The model has been updated and we have new pairs of dispensing errors we’d like you to score.

This survey is just like the first survey you completed. You will see the drug that was prescribed and the drug that was dispensed. On a scale of 0 to 3, we’d like you to rate the severity of the error. 0 indicates no harm is likely to happen to the patient. 3 indicates severe harm is likely (life-threatening, hospitalization, or disability).

# Closing

Wonderful - We will again incorporate the things we have learned today so that we can build a prototype that we can then use to conduct our first pilot of the tool. In the next few months, we will build out the testing platform that will allow us to simulate the verification process and test the effects of these different AI components on pharmacist decision-making and work performance. In the 3rd session, we will demo the platform and gather your feedback for refining the prototype design. We will also ask you to help us define the examples of verification tasks that our pharmacists will perform during the experiment. Be on the lookout for Brigid to schedule this upcoming meeting.
