# Supplementary material for: Designing Human-Centered AI to Prevent Medication Dispensing Errors: Focus Group Study With Pharmacists
Source: JMIR Form Res. 2023 Dec 25;7:e51921. doi: 10.2196/51921 (PMC10775023; doi:10.2196/51921)
Supplement: Multimedia Appendix 3 [file formative_v7i1e51921_app3.docx]

**NIH NLM Images study - Wave 1, Session 3**

**University of Michigan**

**Summer 2022**

# agenda

1. Intro - 5 minutes
2. Model design/discussion - 30 minutes
   1. Bayesian model
   2. New results
   3. Threshold concepts
3. Pick and place designs - 20 minutes (8-10 for designing, 10 minutes for sharing)

[break, 5 minutes]

1. Experimental design - 20 minutes
2. Labvanced examples - critique of the interface, trial design, how realistic, other things that could be included [15 minutes]
3. Conditions of experiment [15 minutes]
4. Pill substitution [10 minutes]

# Introduction/Welcome

## Introduction [5 minutes]

Welcome back to the third and final focus group for our study, AI in the Pharmacy. Thank you for taking the time to meet with us again. To start today’s session, I’d like to briefly review the purpose of these focus groups and the ground rules.

The goal of this study is to develop a novel technology designed to safely and effectively support pharmacy staff in their work of dispensing prescription medications correctly to patients. In this phase of the project, we are asking you to participate in the design sessions. In these sessions, you will complete interactive design activities, respond to questions, and participate in writing activities to help stimulate our discussion. This will help us understand your current work, brainstorm ideas for new tools, and evaluate how those tools might work.

This is intended to be a safe space in which you can talk freely and openly about the topic at hand. There are no right or wrong answers. Given the group setting, we cannot guarantee anonymity of your participation, however, we ask all participants to keep the contents of the discussion confidential and not reveal the identity of your fellow participants to others. As a reminder, this Zoom call will be recorded. If you have any questions before we begin, please feel free to ask them now. [pause] Thanks, I will hit the record button now.

# Model Design and Discussion [30 minutes]

During the first two focus groups we asked for your feedback on the model and its features. We’ve used this feedback to refine our model with the goal of creating an interface that is useful for pharmacists while working within the parameters of the project and the current limitations of the technology.

Today we would like to show you the third iteration of our model and get your feedback. Before we do that, I will give a presentation on the deep learning model we are using for our AI.

## Corey’s Presentation

Reminder of the task

How do DL models work for image data?

Review CNN and Bayesian DL

Compare CNN and Bayesian DL

How to use model output (pred prob and entropy) to demonstrate highly automated system

## Model Thresholds

One important factor that affects the model’s verification performance would be the threshold set for automatically approving a medication. As you may recall from our last meeting, the model outputs are a probability distribution over different NDCs. In other words, for each NDC the network evaluates the likelihood that the inputted image is the specific NDC.


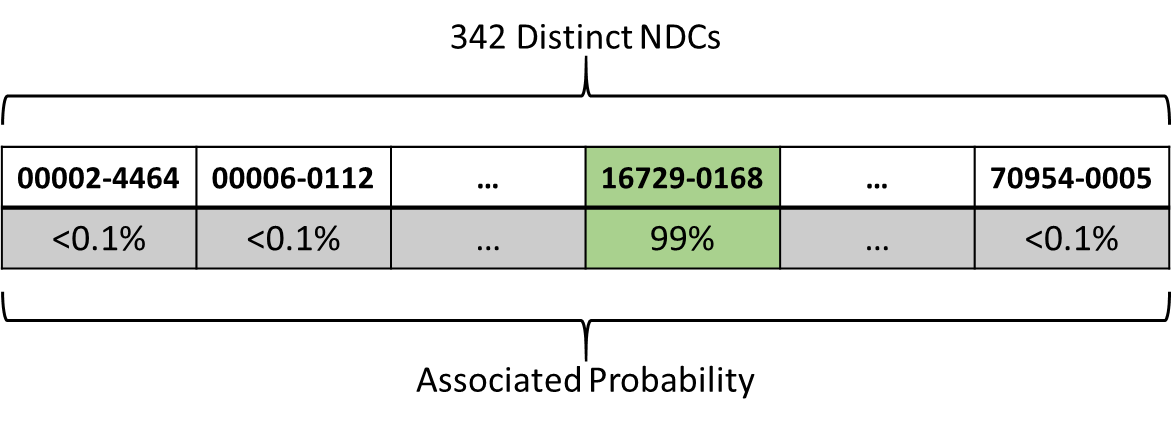


In the case shown here, because the image comes from this NDC the likelihood of that corresponding NDC is very high, close to 100%, while others are close to zero. The model’s job is to pick the NDC with the highest probability or likelihood. We pick the one with the highest likelihood. We can set a threshold for the model based on the probability. The threshold will determine when a pharmacist needs to review a prescription and when it can be automatically approved. For example, if the threshold is set at 95%, anytime the probability is 95% or greater, the prescription will automatically be approved. The pharmacist will not be asked to review the prescription.

In a Bayesian approach, the computer is trained to make several predicted probability distributions and then averages all of the predicted probabilities and selects the NDC with the greatest mean. Because we have several runs of the model, we are able to summarize the distributions into an Entropy score.

Entropy gives us some additional information about the uncertainty of a prediction from the model. Entropy is the amount of variation in the predicted probabilities among all classes. It is a measure of how similar the distribution of the predicted probabilities in the Bayesian model are. The lower the entropy the more certain. Here is an example of the Entropy scores when the AI was correct and incorrect in its prediction:


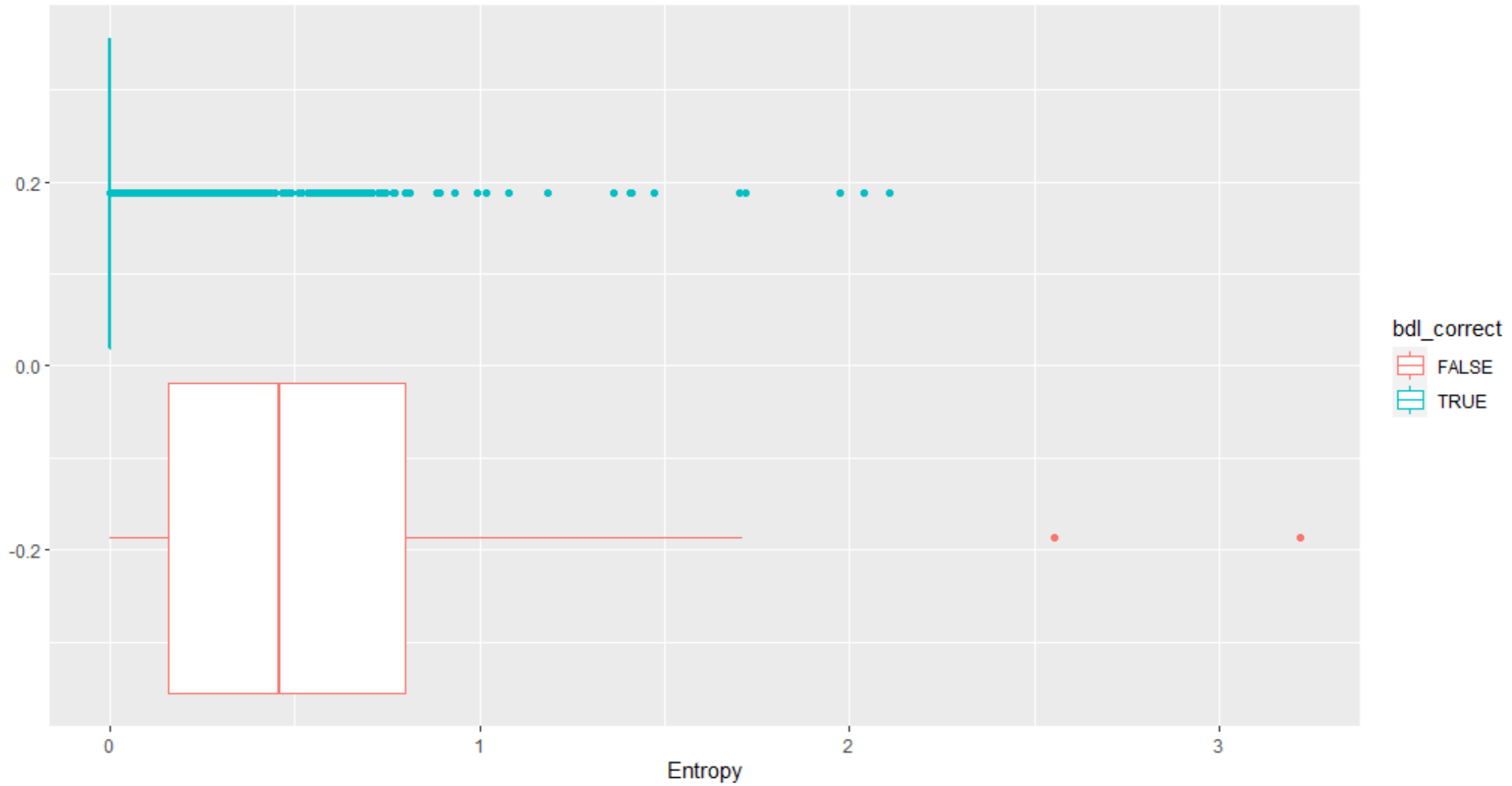


Fig. bdl entropy split by correct and incorrect predictions

- *What do you notice about the two boxplots?*
  - *What differences exist? What similarities?*
- *How might you use Entropy to support the verification of medication?*
- *What else would you want to know about the model?*

What if you could set the threshold for the model, what would it be? Remember, the threshold will determine when you see a prescription and when you won’t. I think an example will be most useful. [switch to R program]. In this program, I have a table which is the cases when the model is correct and incorrect as well as entropy values for each image in the test set (N = 86401). We can use the entropy score to decide when an image could be automatically approved and when an image could be referred for human review. If we summarize the data in a 2x2 table with the correctness of the AI as rows and the acceptance of the image based on entropy values as the columns, we can see how workload and risk changes.

|  | **System approves** | **System sends for review** |
| --- | --- | --- |
| **AI correct** | Good | Unnecessary |
| **AI incorrect** | Problem | Caught error |

- What threshold should we use?
- *What are the benefits and/or consequences of each box?*

Now, let’s use this R program to simulate the distribution of each outcome when changing the Entropy threshold.


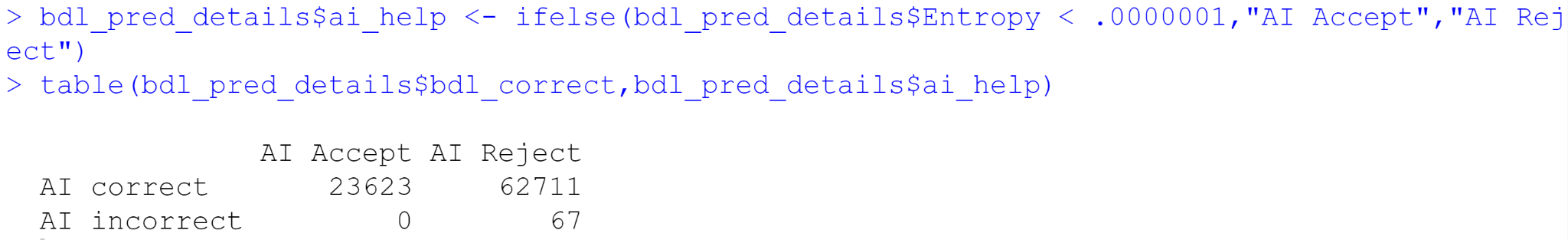


- Based on a threshold of [*insert pharmacist’s threshold]*:
  1. This is the number of times you wouldn't have seen the error
  2. This is the number of times you would have had to review a prescription when there wasn't an error.
- What are your thoughts about the threshold?
- What concerns do you have?
- Are there certain classes of drugs you wouldn’t feel comfortable using this threshold? Why or why not?
- Are there specific situations/drug classes that you would feel comfortable with a lower threshold? Why or why not?

# Pick and Place Design Activity

We have multiple options for how the AI model output can be communicated to pharmacists. I am going to walk you through some potential design elements. After my explanation, I will ask you to break into small groups of 2 or 3 people and allow you to design your own interface using the elements we discussed.

## Discussion of Element

1. Accept / reject / not sure
2. NDC predicted
   1. Does shape, color, size, etc match the NDC reference?
3. Predicted probability
   1. Value itself
   2. Value in terms of the range of possibilities
   3. Gradient scale showing where the value falls in the range of possibilities
4. Standard deviation or Entropy
   1. Value itself
   2. Value in terms of the range of possibilities
   3. Gradient scale showing where the value falls in the range of possibilities
5. Machine’s accuracy on the true_label class
   1. and where it ranks

## Participant Design

I’m sending each of you a link to a Google Drive file with all the elements we just discussed. In a moment, I’ll place you in a Zoom breakout room with 1-2 other people. You’ll spend approximately 8-10 minutes designing the user interface you would like to see. You can use as many of the elements as you would like. Think about where you would like the elements placed to make the verification process as easy as possible. There are no constraints. After the 8-10 minutes are up, we’ll regroup and I’ll ask each small group to present their user interface. What questions do you have?

Great. I’ll put you in the breakout rooms. The breakout rooms will close at [X:XX]

## Presentation of Design

I would like each group to present their design. Who would like to go first?

*Potential prompts*

- *What is the most important element for you? Why?*
- *Can you discuss the placement of that element? Why is it important for the element to be located in that position?*
- *Tell me a bit about why you didn’t use X element*
- *What aspects of the system successfully present information?*
- *What are ways you’d like this system to be improved to support information presentation?*

# 5-minute Break

Thanks for all the great input on the interface design! We’ll take a 5-minute break now and resume our discussion at [X:XX].

# Experimental Design [30 minutes]

Welcome back. Now let’s discuss how our model and user interface will be used. In the next phase of the project, we will recruit pharmacists to participate in a series of 100 remote verification tasks. Each pharmacist will receive a 15-minute self-guided training session that introduces the study and teaches participants how to use the interface and perform the medication verification task. In each task, participants will compare the image on the left (i.e., the medication filled for a prescription) to the image on the right (i.e., a known reference image) and decide whether to accept or reject the images as containing the same prescribed medication. All of this will be done using a software program called, LabVanced.

I will now go through the software that we’ll be using to conduct this experiment. I have a couple of short videos for you to watch to see how the experiment will be set up and how the users will answer questions. I would like you to think about items like providing a critique of the interface (ie., how does it compare to the software you use, how can it be made more realistic/prototypical), drawbacks of the trial design, and how realistic it is for the pharmacists to complete these tasks.

# Model Outputs by Condition [15 minutes]

In Wave 2 of the project, there are 4 possible conditions pharmacists may see. By conditions I mean how the interface will display information. The 4 possible conditions are:

1. No help: No help is pretty self-explanatory. No AI will be involved and pharmacists will perform the verification without any AI assistance.
2. AI help with advice
3. AI help with uncertainty: As we just finished discussing entropy shows how variable the probability estimates were. It shows how much certainty there is in the predicted medication NDC.

I would like to have a group discussion about how each of these conditions might look to the pharmacists.

**Interface display cases for No MI help:**

**
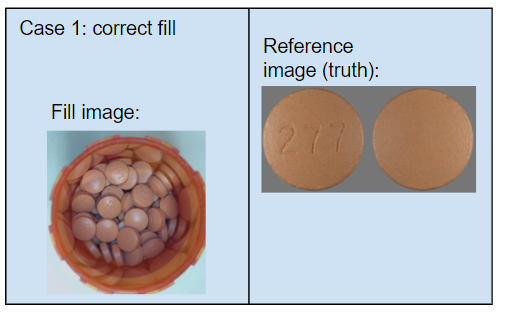

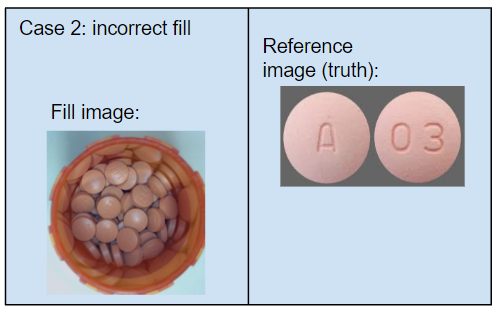
**

**Interface display cases for MI help:**


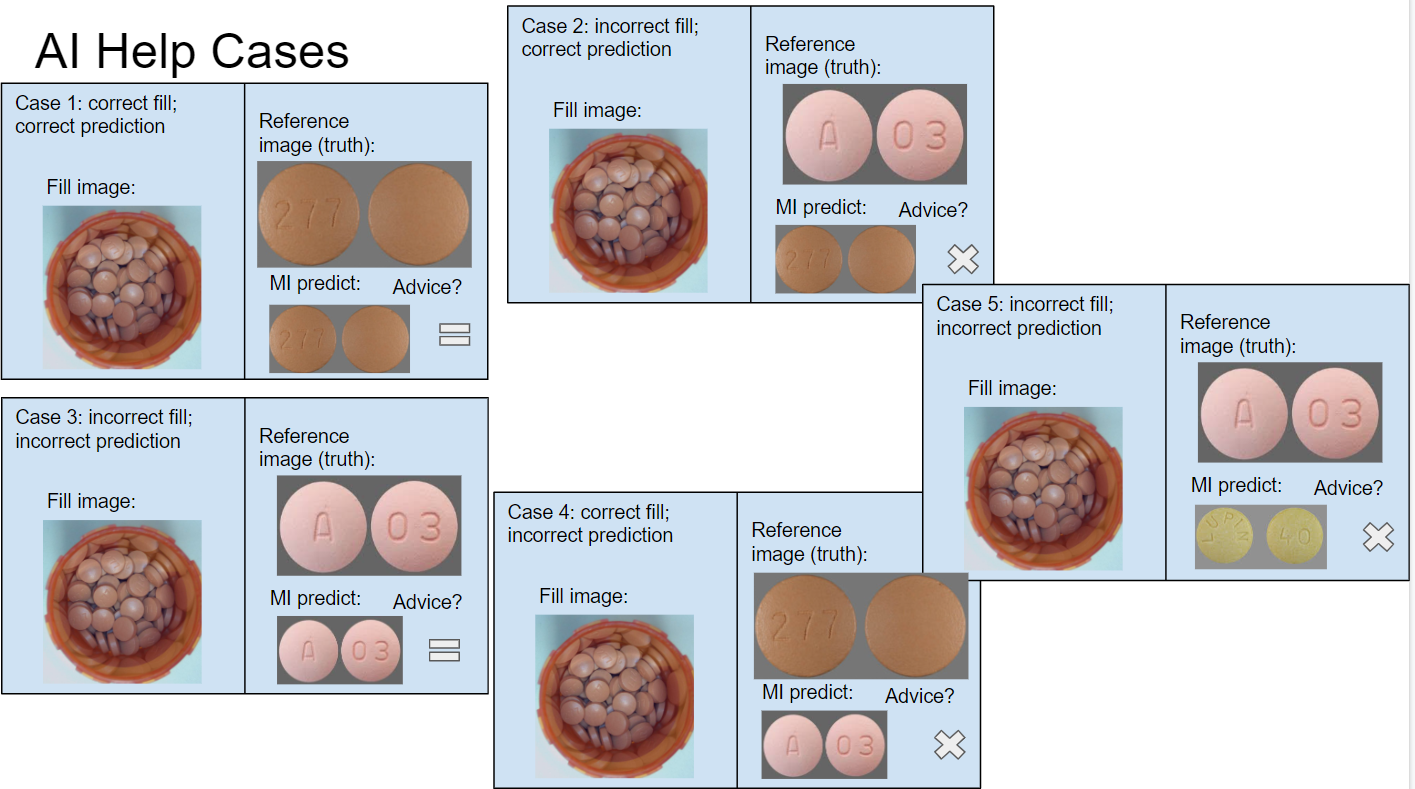


# Pill Substitutes [10 minutes]

As you know, no system is infallible. Last time we showed you some examples of images the model predicted incorrectly. Let’s go back to some of those examples and I’ll ask you to review the images that were incorrectly predicted by the model.

- What are the ones we should substitute here?
- What would be reasonable to 'mispick' / mis-fill?
  - What would not be reasonable?
- What are case examples of misfilled medications that should be included in Wave 2? (eg, medications from the same manufacturer but different strengths, medications with similar size/shapes/colors)
  1. What are pill substitutes (ie., errors) you’ve seen in the pharmacy?
- How often do you encounter misfilled medication that requires you to send the prescription back to staff to fill with the correct medication? Please respond in terms of a natural frequency (eg., 1 in 100)
- What are some reasons that you might miss the correct medication being dispensed? (eg, noisy/distracted environment, too many other things to check)

Now I’m sending you a link to a Google Drive file. When you open it, you’ll see a spreadsheet with images of pills. These are examples of images the model predicted incorrectly. In each row, the first image is an image of the true pills and the associated NDC details. Next you’ll see the incorrect model prediction and its associated details. For each row, we’d like you to do three things:

1. Respond yes or no to whether you can tell the difference between the two images
2. Indicate if this is a filling error you might expect to see. Provide brief details on why or why not.
3. Indicate the severity of the error if the patient received it.


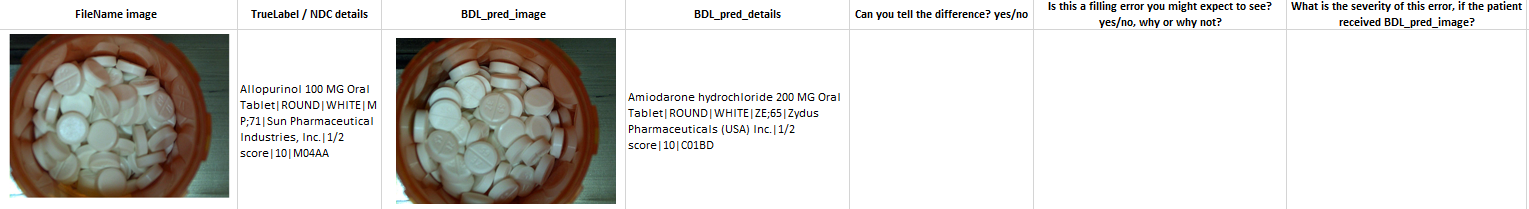


Dropbox (University of Michigan)\Images Study\Focus Group_BDL errors_2022.06.13.xlsx

# Closing

Wonderful - this is great feedback. As you know, this is our last focus group meeting. Before we close the session, are there any final questions or comments?

[Pause. Answer any questions.]

It has been great getting to know all of you and I want to thank you again for taking the time to participate in this research project. Your feedback has helped us tremendously. Have a great evening.
